# Supplementary material for: Oleiferasaponin A2, a Novel Saponin from Camellia oleifera Abel. Seeds, Inhibits Lipid Accumulation of HepG2 Cells Through Regulating Fatty Acid Metabolism
Source: Molecules. 2018 Dec 12;23(12):3296. doi: 10.3390/molecules23123296 (PMC6321182; doi:10.3390/molecules23123296)
Supplement: Supplementary file 1 [file molecules-23-03296-s001.pdf]

SUPPLEMENTAL MATERIAL

Supplemental Figure 1. HPLC spectrum of oleiferasaponin A_2

Supplemental Figure 2. IR spectrum of oleiferasaponin A_2

Supplemental Figure 3. Mass spectra of oleiferasaponin A_2

Supplemental Figure 4. ^{1}H-NMR spectrum of oleiferasaponin A_2

Supplemental Figure 5. ^{13}C-NMR spectrum of oleiferasaponin A_2

Supplemental Figure 6. COSY spectrum of oleiferasaponin A_2

Supplemental Figure 7. HSQC spectrum of oleiferasaponin A_2

Supplemental Figure 8. HMBC spectrum of oleiferasaponin A_2

Supplemental Figure 9. NOESY spectrum of oleiferasaponin A_2

Supplemental Figure 10. GC-MS spectrum of oleiferasaponin A_2

Supplemental Table 1. Primer sequence

Supplemental Figure 1. HPLC spectrum of oleiferasaponin A_2

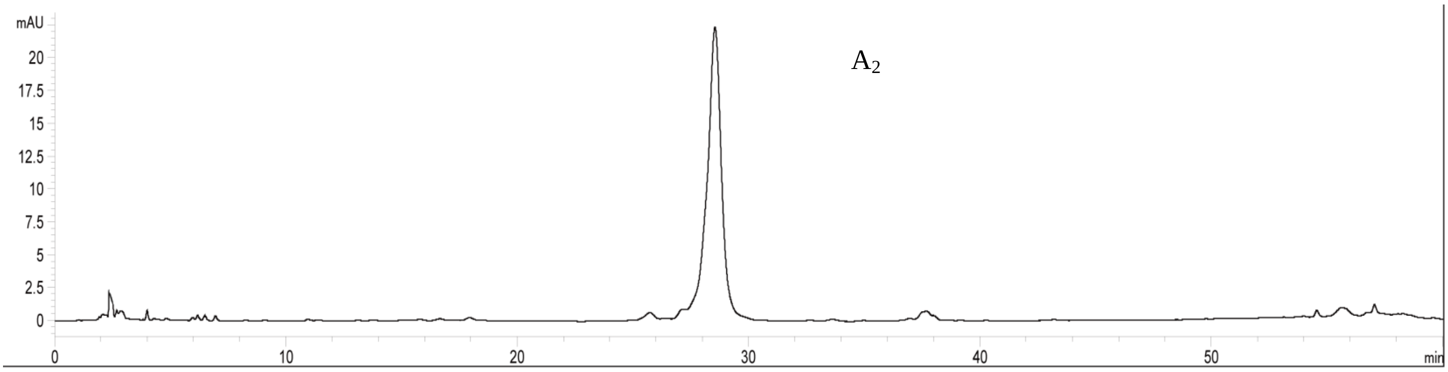

Supplemental Figure 2. IR spectrum of oleiferasaponin A_2

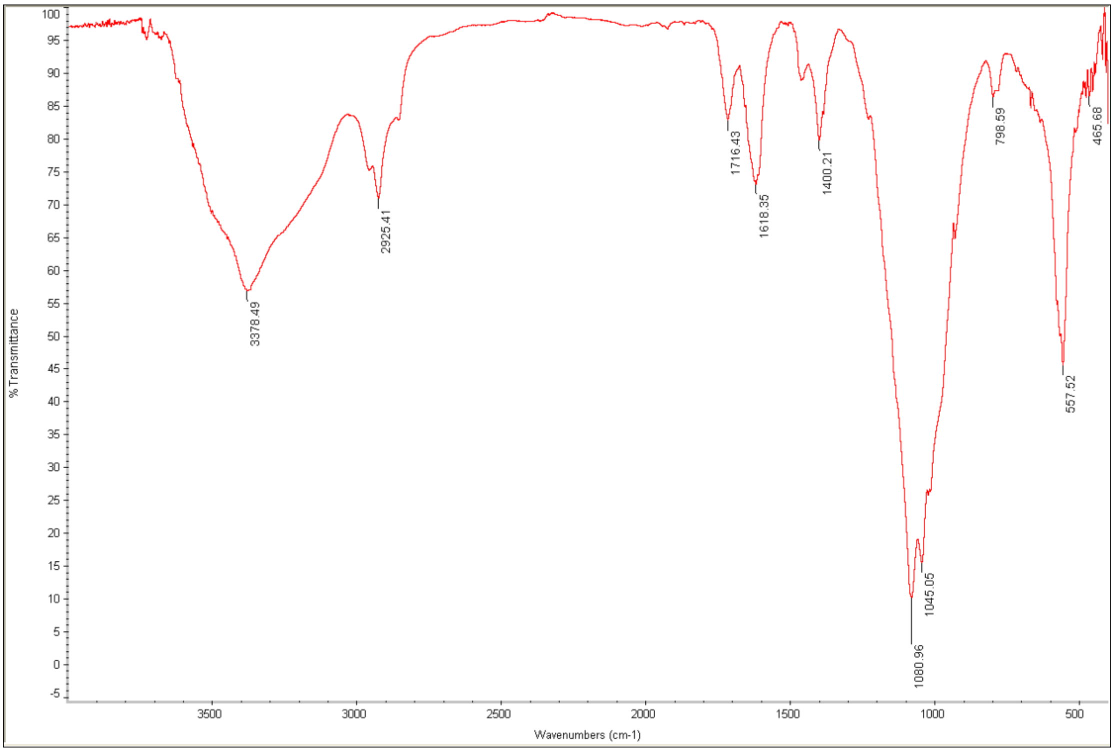

Supplemental Figure 3. Mass spectra of oleiferasaponin A_2

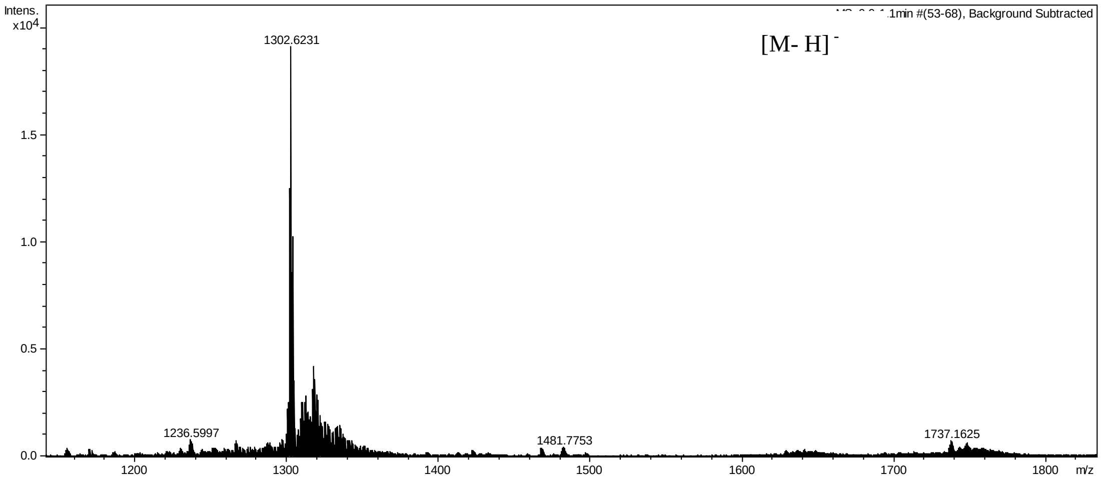

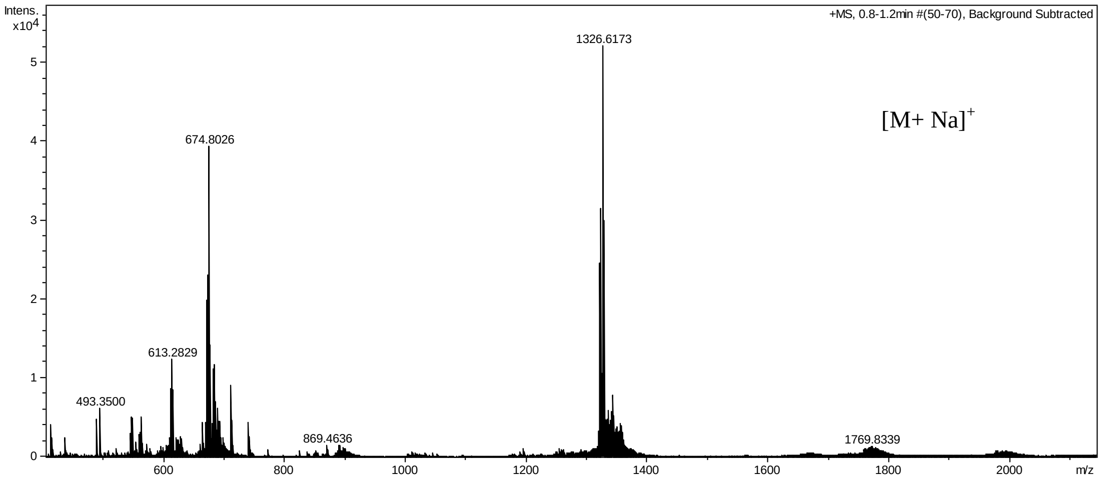

Supplemental Figure 4. ^{1}H-NMR spectrum of oleiferasaponin A_2

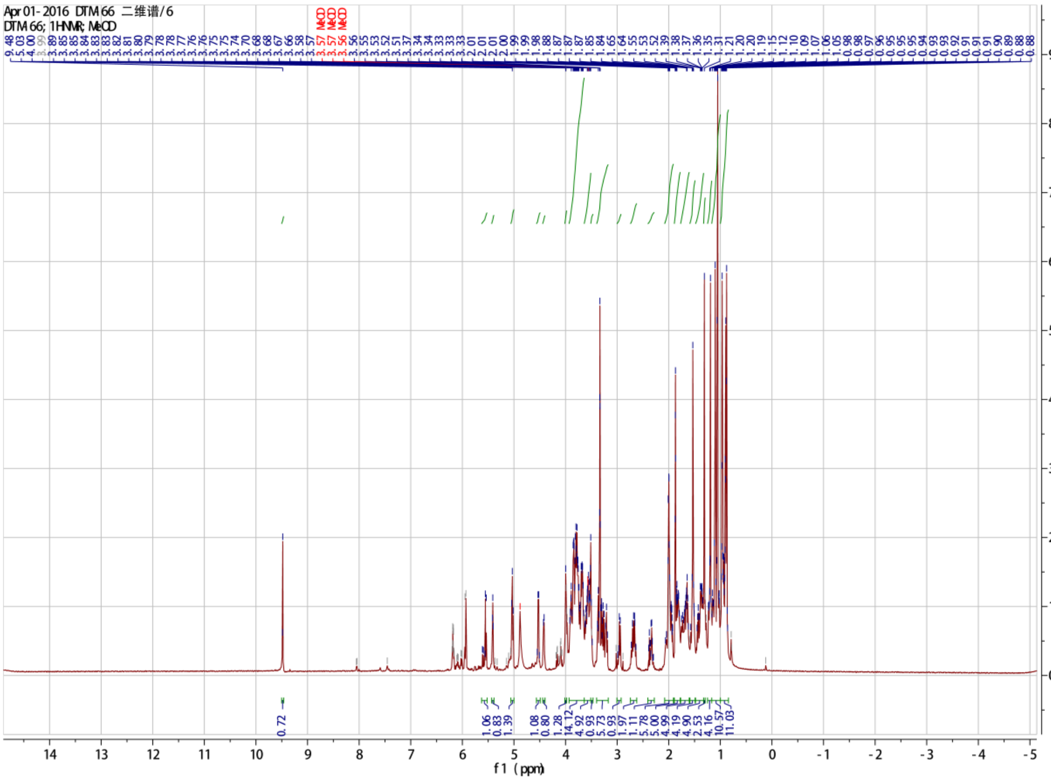

Supplemental Figure 5. ^{13}C-NMR spectrum of oleiferasaponin A_2

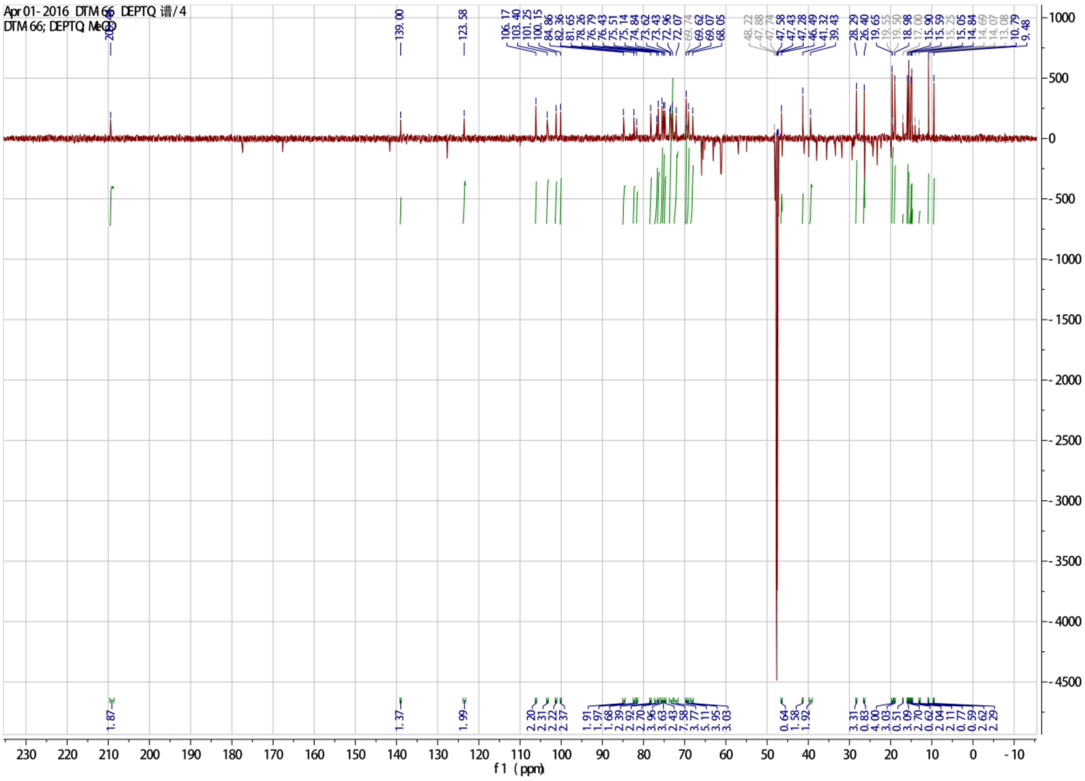

Supplemental Figure 6. COSY spectrum of oleiferasaponin A_2

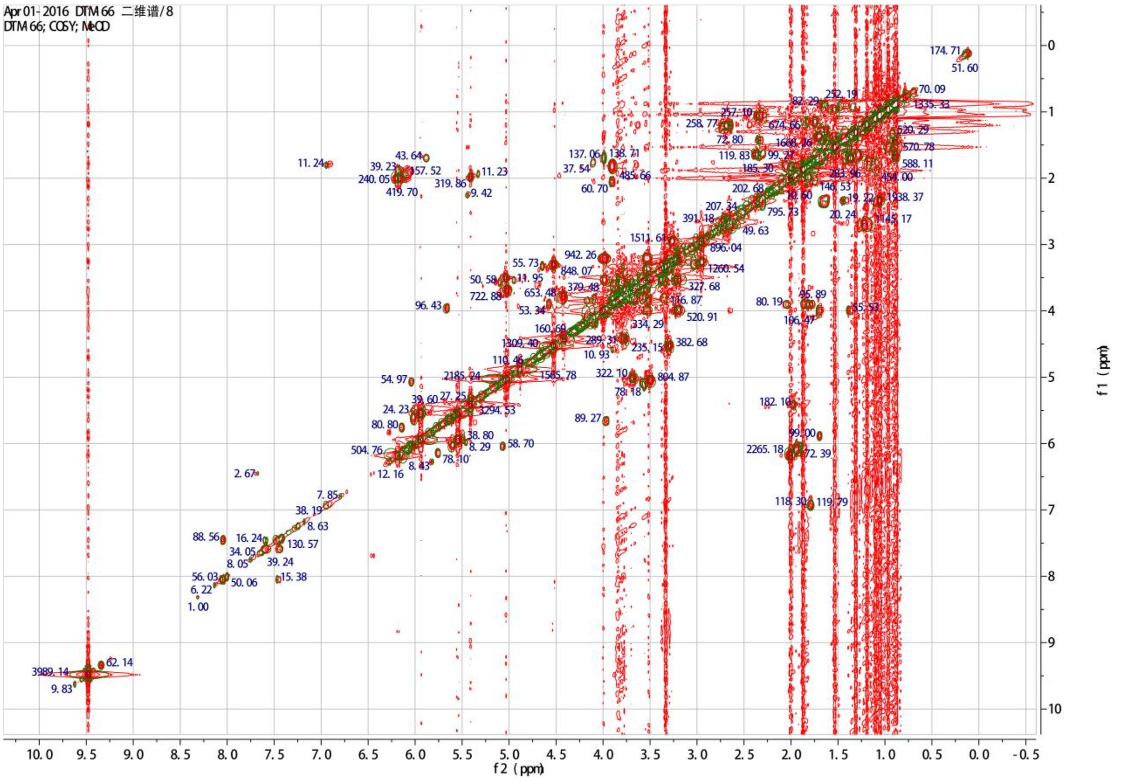

Supplemental Figure 7. HSQC spectrum of oleiferasaponin A_2

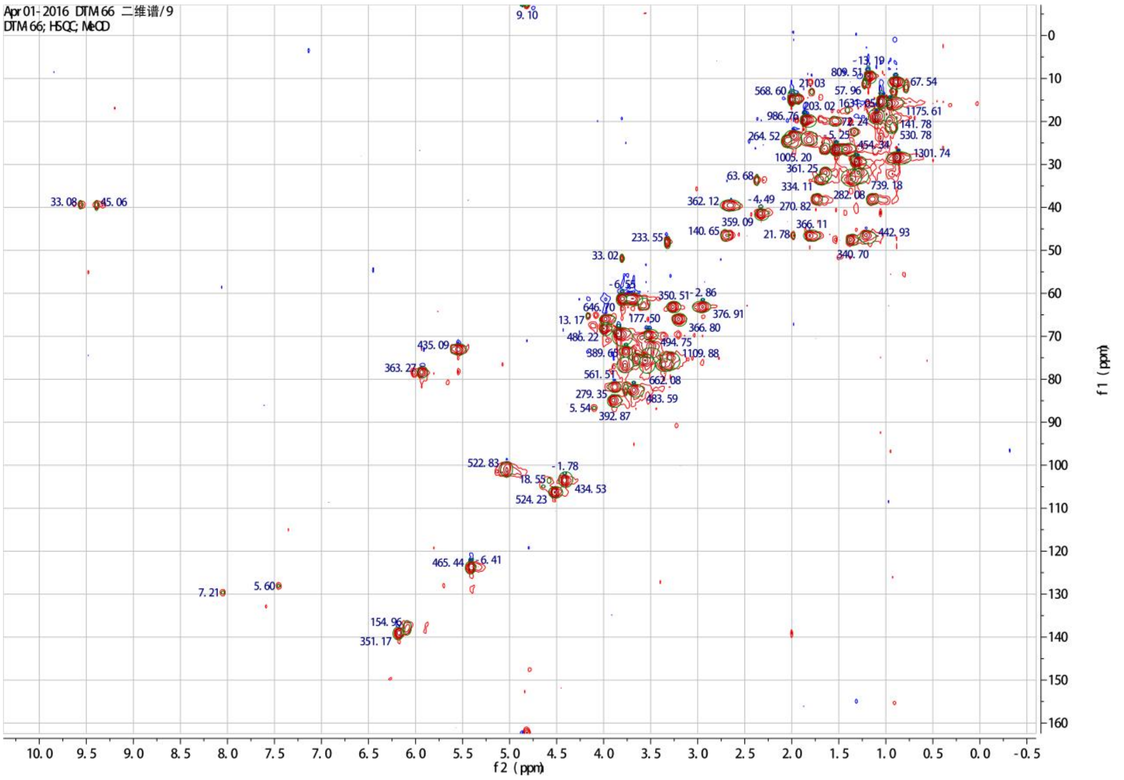

Supplemental Figure 8. HMBC spectrum of oleiferasaponin A_2

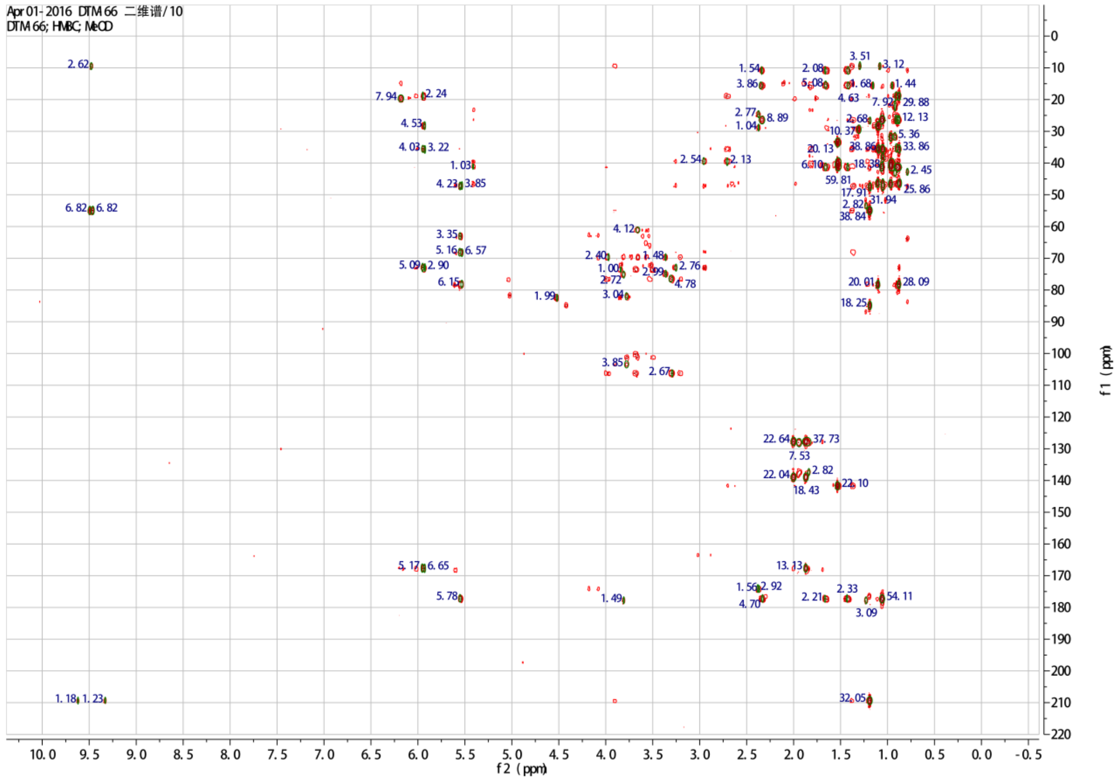

Supplemental Figure 9. NOESY spectrum of oleiferasaponin A_2

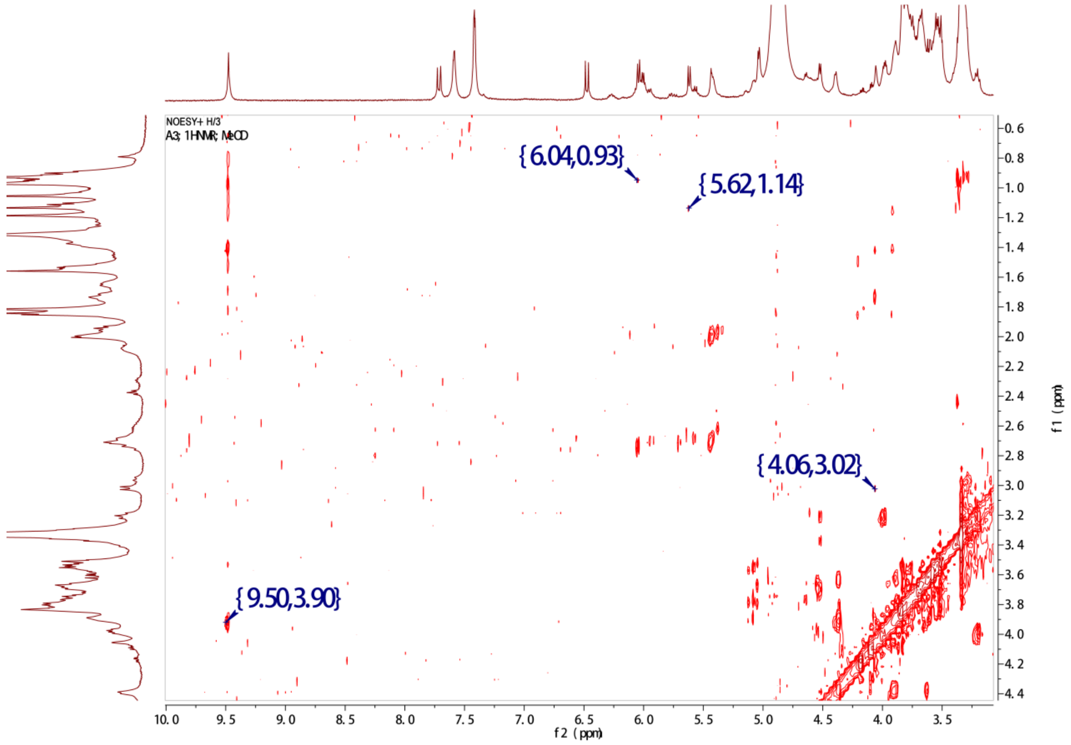

Supplemental Figure 10. GC-MS spectrum of oleiferasaponin A_2

1. L-Ara

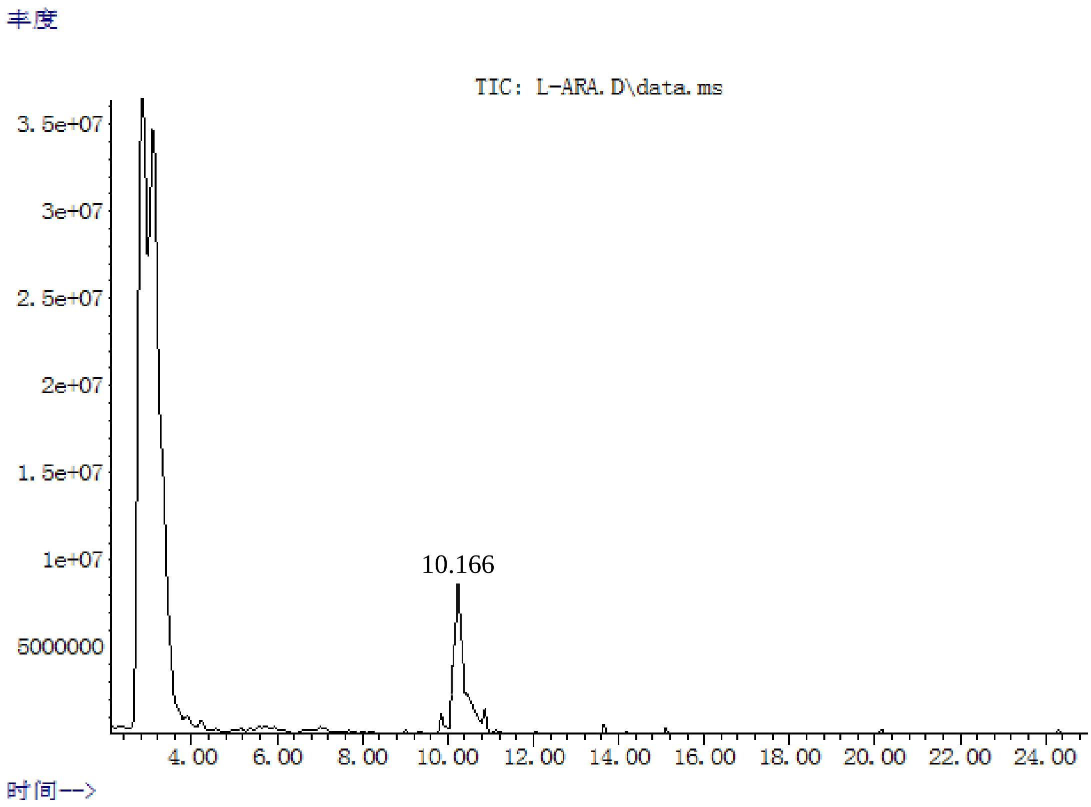

2. D-Glu

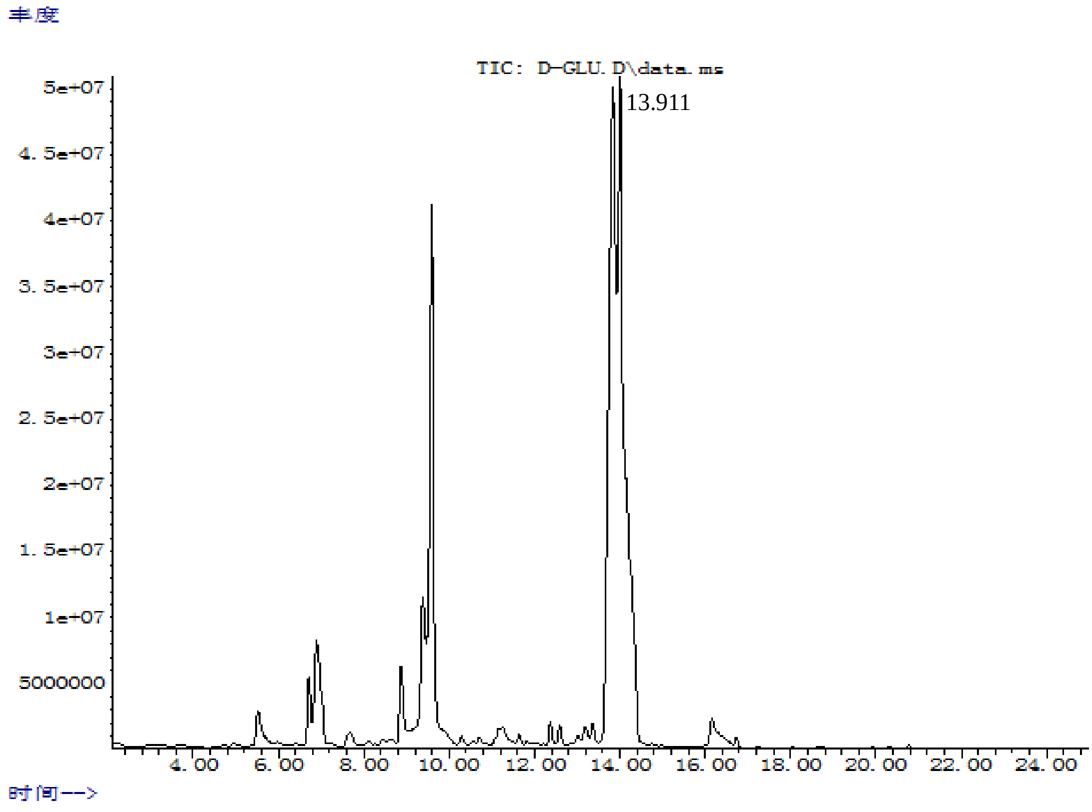

3. D-Gal

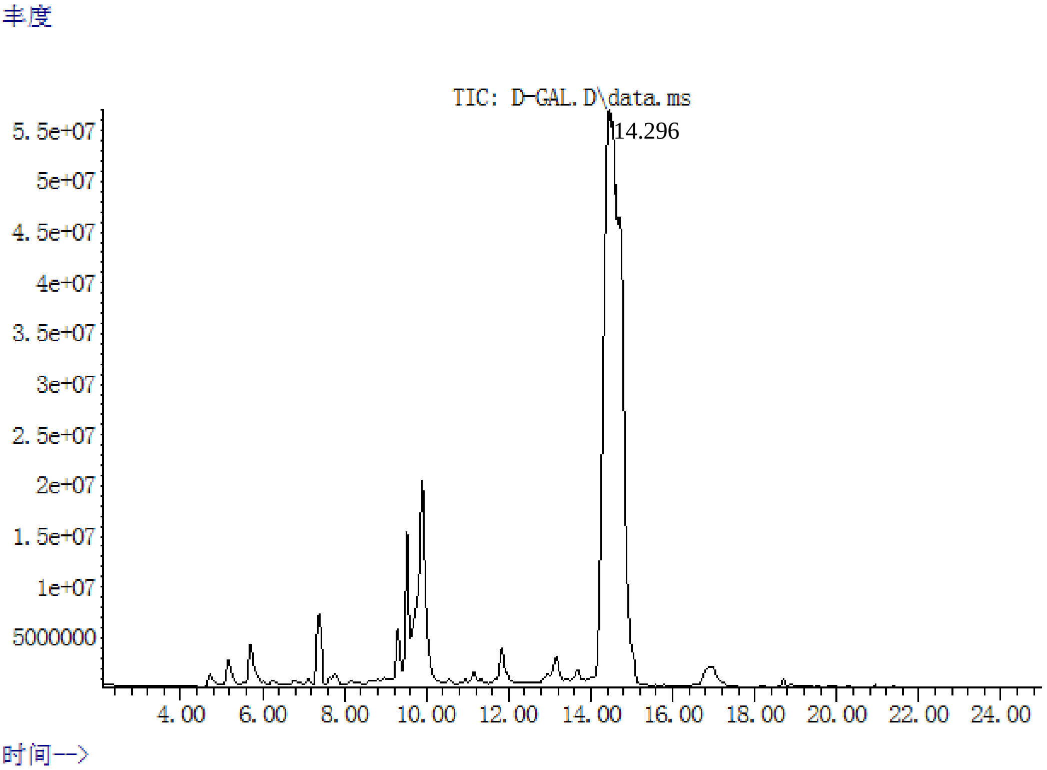

4. **Oleiferasaponin A_2**

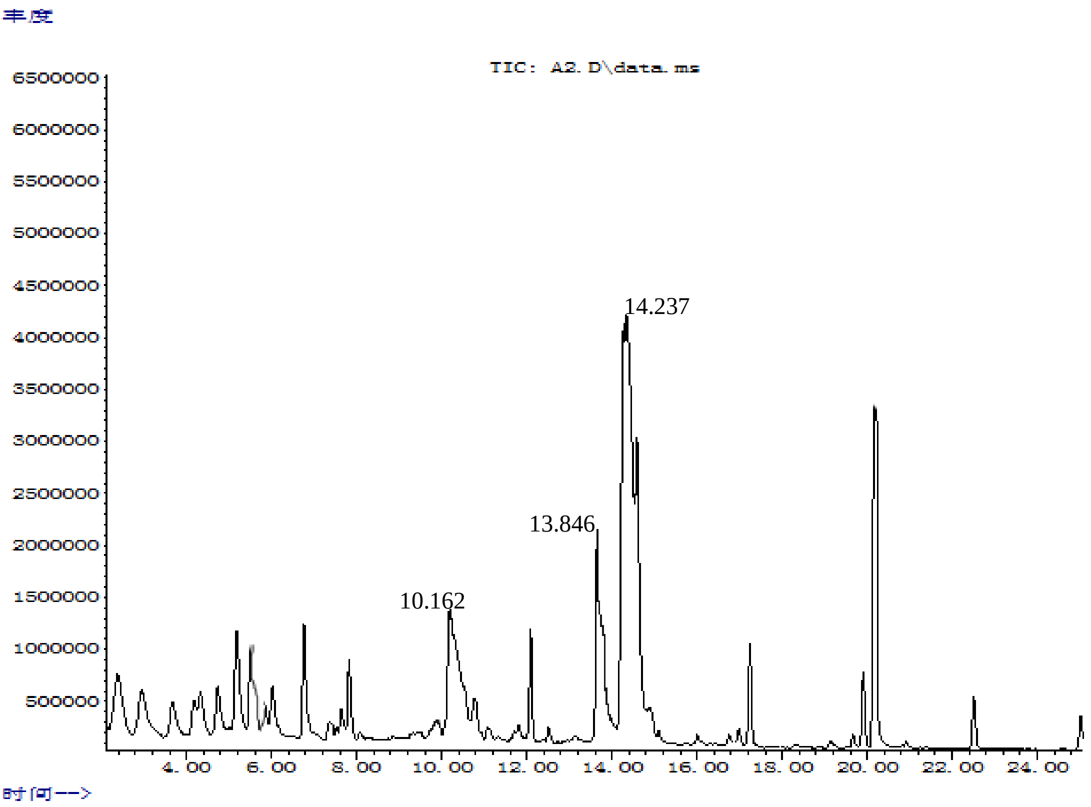

Table 1. Primer sequence.

Primer	Sequence (5'−3')
ACC-F	TGATGTCAATCTCCCCGCAGC
ACC-R	TTGCTTCTTCTCTGTTTTCTCCCC
PPARα-F	AAAAGCCTAAGGAAACCGTTCTG
PPARα-R	TATCGTCCGGGTGGTTGCT
SREBP-1c-F	CCATGGATGCACTTTCGAA
SREBP-1c-R	CCAGCATAGGGTGGGTCAA
FAS-F	CGGTACGCGACGGCTGCCTG
FAS-R	GCTGCTCCACGAACTCAAACACCG
ACOX1-F	GGGCATGGCTATTCTCATTGC
ACOX1-R	CGAACAAGGTCAACAGAAGTTAGGTTC
CPT1-F	CGTCTTTTGGGATCCACGATT
CPT1-R	TGTGCTGGATGGTGTCTGTCTC
GAPDH-F	AGGAGGCATTGCTGATGATC
GAPDH-R	GTCTTCACCACCATGGAGAA
